# Effect of Physical Nature (Intact and Powder) of Coal on CO_2_ Adsorption at the Subcritical Pressure Range (up to 6.4 MPa at 298.15 K)

**DOI:** 10.1021/acsomega.2c07940

**Published:** 2023-02-10

**Authors:** Maram Almolliyeh, Snehasis Tripathy, Sivachidambaram Sadasivam, Shakil Masum, Hywel Rhys Thomas

**Affiliations:** Geoenvironmental Research Centre (GRC), School of Engineering, Cardiff University, The Queen’s Buildings, The Parade, Cardiff, CF24 3AA, United Kingdom

## Abstract

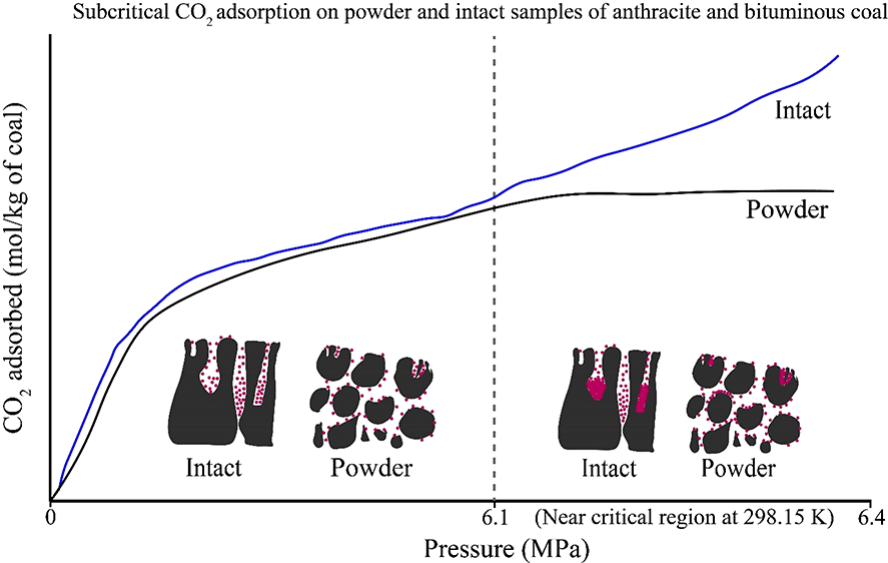

This study examines the influence of subcritical pressure and the physical nature (intact and powder) of coal samples on CO_2_ adsorption capacity and kinetics in the context of CO_2_ sequestration in shallow level coal seams. Manometric adsorption experiments were carried out on two anthracite and one bituminous coal samples. Isothermal adsorption experiments were carried out at 298.15 K in two pressure ranges: less than 6.1 MPa and up to 6.4 MPa relevant to gas/liquid adsorption. The adsorption isotherms of intact anthracite and bituminous samples were compared to that of the powdered samples. The powdered samples of the anthracitic samples had a higher adsorption than that of intact samples due to the exposed adsorption sites. The intact and powdered samples of bituminous coal, on the other hand, exhibited comparable adsorption capacities. The comparable adsorption capacity is attributed to the intact samples’ channel-like pores and microfractures, where high density CO_2_ adsorption occurs. The adsorption–desorption hysteresis patterns and the residual amount of CO_2_ trapped in the pores reinforce the influence of the physical nature of the sample and pressure range on the CO_2_ adsorption–desorption behavior. The intact 18 ft AB samples showed significantly different adsorption isotherm pattern to that of powdered samples for experiments conducted up to 6.4 MPa equilibrium pressure due to the high-density CO_2_ adsorbed phase in the intact samples. The adsorption experimental data fit into the theoretical models showed that the BET model fit better than the Langmuir model. The experimental data fit into the pseudo first order, second order, and Bangham pore diffusion kinetic models showed that the rate-determining steps are bulk pore diffusion and surface interaction. Generally, the results obtained from the study demonstrated the significance of conducting experiments with large, intact core samples pertinent to CO_2_ sequestration in shallow coal seams.

## 1 Introduction

Geological carbon dioxide (CO_2_) storage, especially, sequestration in unmineable coal seams, has a high potential owing to higher CO_2_ storage capacity.^1,2^ Numerous experimental studies have revealed that coal can adsorb and store CO_2_, and coal seams have the potential to store 300–964 Gt CO_2_ globally.^1−6^ CO_2_ storage capacity of coal seams are estimated by considering the experimentally determined adsorption capacity of the various coal ranks as one of the factors.^1^ However, the knowledge gap in the effect of sample physical type (intact or powdered) and subcritical pressure range (liquid and gas) on CO_2_ adsorption must be improved in order to comprehend the storage potential of the shallow level coal seams.

Adsorption capacity and kinetics of CO_2_ adsorption on coal samples are often related to coal rank, moisture content, swelling characteristics, porosity, temperature, and operating pressures.^1,7−9^ In general, the CO_2_ adsorption capacity of a specific rank of coal increases with pressure and showed decreasing trend in a few studies.^10−15^ To maximize storage capacity and safety, supercritical CO_2_ injection into coal seams deeper than 500 m has been considered. However, at greater depths, the confining pressure and coal swelling influence the permeability of high-density CO_2_ and hinder the storage potential.^16^ As a consequence, CO_2_ storage at shallower depth coal deposits has received attention, and horizontal injection of subcritical CO_2_ in existing shallow level coal mines has been regarded as a technological advance because it improves the CO_2_ contact area while reducing the impact of coal swelling.^17^ CO_2_ adsorption is a gas/liquid phase adsorption at coal seam depths where the temperature and pressure values are less than the critical value (31 °C and 7.38 MP).^18−21^ The density of CO_2_ is extremely sensitive near the critical point (van der Waals loop region) where the coexistence of liquid and vapor phases would influence the way coal-CO_2_ interaction occurs in general.^14,22^

Adsorption capacity and kinetics data on intact coal samples are limited for the subcritical range^20,23−25^ due to the difficulties with core sample extraction, extended time needed to achieve equilibrium, variable permeability, and pore diffusion/condensation.^24^ Moreover, pulverizing coal samples can alter or lose their physical nature, which affects their CO_2_ adsorption properties.^26−28^ Consequently, it stands to reason that comparing the CO_2_ adsorption capacity of intact and powdered samples of various coal ranks would reveal the effect of the sample’s physical type on CO_2_ adsorption. Therefore, more laboratory investigations with intact coal samples at subcritical temperature and pressure region are fundamental to understanding coal-CO_2_ interactions.

Methane desorption was widely focused on in previous works,^29−31^ the CO_2_ desorption data are less discussed in the literature, and it is important to investigate the reversibility of pore trapped CO_2_. The pore trapping mechanisms such as pore blockage, gas cavitation, adsorption induced deformation, and pore network (ink bottle effect) affect the adsorption–desorption hysteresis pattern and correlate with the coal rank.^32^ In general, a limited number of studies modeled the desorption kinetics.^32,33^ Among the two dominant kinetic models, pseudo-first order (PFO) and pseudo-second order (PSO), the PSO model agreed well with the experimental results obtained using a manometric adsorption experimental set up for an intact and powdered bituminous coal sample, implying that CO_2_ adsorption kinetics and hysteresis were determined by pore diffusion and condensation.^34,35^ Njikam and Schiewer (2012)^36^ modified the commonly used adsorption kinetic models (PFO and PSO) to adopt the desorption process. These models have not been fully explored for CO_2_ adsorption–desorption.^23^ The rate-determining steps for the adsorption–desorption process can be predicted by fitting the adsorption–desorption kinetics experimental data in the Bangham pore diffusion model and the modified PFO and PSO equations.^36,37^

While efforts have been made to understand supercritical CO_2_ adsorption on powdered samples, the present study seeks to investigate the CO_2_ adsorption capacity and kinetics of intact anthracite and bituminous coal samples in comparison to powdered samples, how the adsorption–desorption isotherm hysteresis differs for powder and intact samples in terms of the physical type of coal, and the effects of subcritical and the near critical injection pressure range at 298 K. For this, a manometric gas adsorption experimental setup was employed. The results of adsorption studies utilizing intact and powdered samples of two anthracite coals and a bituminous coal were fitted into theoretical isotherm and kinetic models to determine the CO_2_ adsorption mechanism at 298.15 K.

## 2 Materials and Methods

### 2.1 Coal Properties and Sample Preparation

Coal samples were collected from two coal mines in South Wales Coalfield, Wales, UK. The anthracite coal samples were obtained from the Aberpergwm coal mine (51°44′28.8″N 3°38′36.0″W), while the bituminous coal (water content 0.96%) samples were obtained from the Big Pit National Museum (51.7724°N 3.1050°W). Coal blocks were extracted from the Aberpergwm colliery’s two coal seams, the 9 ft seam (with a water content of 0.91%) at a depth of 550 m and the 18 ft seam (water content 0.78%) at a depth of 500 m. The Big Pit coal had a water content of 0.96% and was extracted from a coal seam at a depth of 90 m. These samples will be referred to as 9 ft AB and 18 ft AB and BP hereafter.

The proximate and ultimate analyses were conducted in accordance with the British Standards Institution (BSI) and American Society for Testing Materials (ASTM) standards.^38−43^Table 1 summarizes the analyses. On the basis of carbon content, volatiles, and gross calorific value, the Aberpergwm samples (9 ft AB and 18 ft AB) were classified as anthracite coal (high rank). The Big Pit (BP) sample was identified as low volatile bituminous coal based on their carbon content.^44,45^

**Table 1:** Proximate and Ultimate Analysis of the Coal Samples Investigated in This Study

analytical		18 ft AB	9 ft AB	BP
Proximate analysis				
Water Content	% mass	0.78	0.91	0.96
Ash Content	% mass	1.38	4.62	12.7
Volatiles content	% mass	5.08	5.73	29
Calorimetry				
High calorific value	MJ/kg	35.04	35.60	33.68
Low calorific value	MJ/kg	34.30	32.89	-
Ultimate analysis				
Total Carbon	% mass	92.05	89.5	83.87
Total sulfur	% mass	0.73	0.87	1.62
Total hydrogen	% mass	3.31	3.16	-
Nitrogen	% mass	1.27	1.31	3.3
Oxygen	% mass	0.5	0.33	-

### 2.2 Intact and Powdered Sample Preparation and SEM Imaging

**Figure 1:**
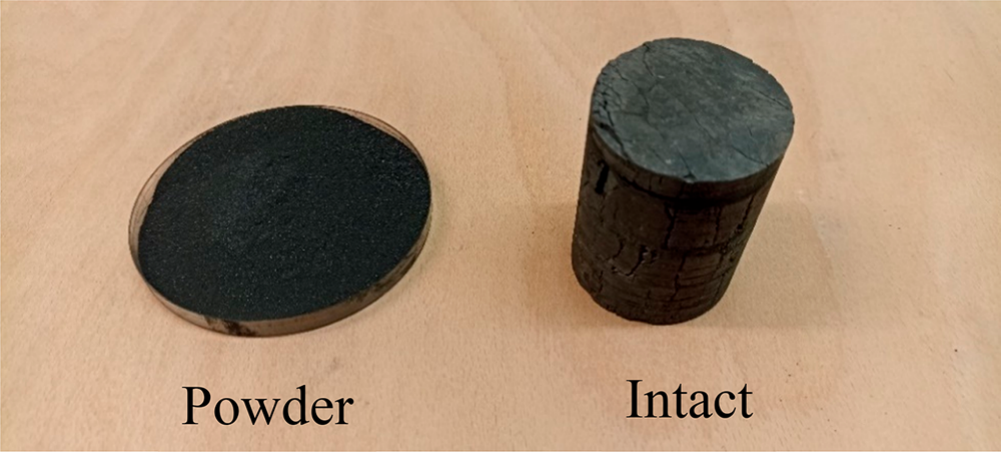
Photographs of coal core and powdered samples.

Coal cores were drilled from large coal blocks using a core drill machine equipped with a diamond saw-tipped core drilling bit with a 50 mm internal diameter. Samples were air-dried prior to conducting adsorption experiments. Figure 1 illustrates the coal core sample and powdered sample. To prepare powdered samples, coal chunks were pulverized and passed through a 63 μm-mesh screen.

The SEM images of the intact and powdered coal samples were obtained using a Zeiss Sigma HD field emission gun analytical high-resolution SEM (natural samples). The samples were finely gold coated to reduce the charging effect, and the images were used to visualize the structural change in the powder and intact samples of relevant coal rank, as discussed in section 4.1.

### 2.3 Methodology

**Figure 2:**
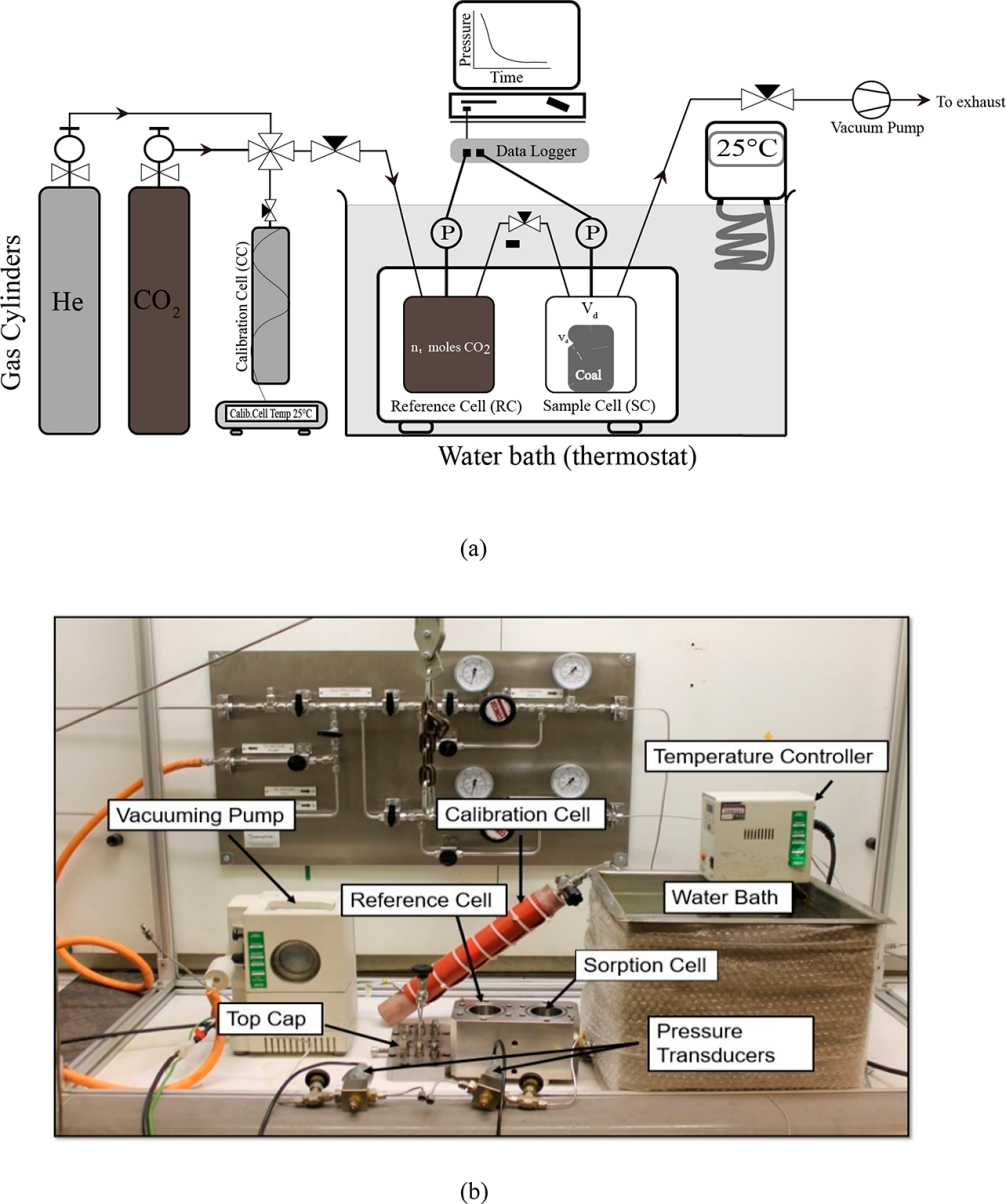
(a) Schematic of the manometric adsorption experimental setup and (b) a photograph of the main components of the manometric adsorption set up.

A manometric adsorption apparatus (volumetric) was used in this study. The apparatus was supplied by GDS Instruments, UK. It is capable of operating at pressures up to 20 MPa and temperatures up to 338 K (65 °C). Schematic of the manometric sorption apparatus and experimental setup is presented in Figure 2a, and a photograph of the major components is presented in Figure 2b. The test setup instrumentation is as follows: (1) a manometric unit consisting of a reference cell (RC) for storing a known quantity of gas and a sample cell (SC) for storing the adsorbent, (2) needle valves for connecting and isolating the RC and SC, (3) pressure transducers with a resolution of 0.002 MPa (2 kPa) and accuracy of 0.15%, and data loggers, (4) a water bath with a temperature controller for maintaining the temperature of 298.15 K ± 0.01 K, and (5) A calibration cell (volume = 0.0004892 m^3^) that determines the empty and sample loaded void volumes of RC and SC using the helium pycnometry (He-pycnometry) method. The calibration cell heater is kept at a constant temperature of 298.15 K ± 0.01.

Manometric adsorption is a mass balance technique that utilizes precise pressure, volume, and temperature measurements.^46^ The experimental concept is as follows: (i) determine void volumes (*v*_d_) of RC and SC using the He-pycnometry method, (ii) prepare a known quantity of CO_2_ gas in RC and expand it into SC while monitoring the pressure drop; repeat the procedure progressively by increasing pressure in RC; and (iii) calculate the adsorbed amount using an appropriate equation of state (EoS) for CO_2_ and the perfect gas law.

The He-pycnometry method, which involves injecting He into the adsorption cell at experimental ambient temperature and pressure, can be used to approximate the void volume (*v*_d_) available for gas molecules in the adsorption cell. The void volume (*v*_d_) is calculated via the He-pycnometry method and using the perfect gas law as^46−50^
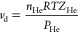
1where *n*_He_ is the number of moles of He injected (mol), *P*_He_ is the pressure of He (Pa), *T* is the absolute temperature (298.15 K), and *R* is the gas constant (8.314 J/mol·K). The compressibility factor (*Z*_He_) values were calculated using the Peng–Robinson equation of state (PR-EoS).^22^

The amount of CO_2_ adsorbed or desorbed is calculated as follows:^46−49^
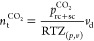
2
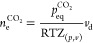
3
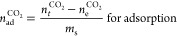
4
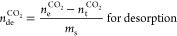
5where *n*_ad/de_^CO_2_^ is the number of moles of CO_2_ adsorbed during adsorption (ad) and desorption (de) (mol; 44.01 g of CO_2_/mol), *n*_*t*_^CO_2_^ is the known amount of CO_2_ present in the gas phase (RC + SC) at the beginning of the adsorption or desorption experiment (mol), *n*_e_^CO_2_^ amount of CO_2_ present in the gas phase (RC + SC) at the equilibrium (end of the adsorption or desorption test) (mol), *v*_d_ is the void volume available for gas (m^3^), *p*_rc + sc_^CO_2_^ is the initial CO_2_ pressure in RC and SC before adsorption, *p*_eq_^CO_2_^ is the equilibrium pressure of CO_2_ in RC and SC after adsorption (Pa), *Z* is the compressibility factor of CO_2_, and *R* is the universal gas constant (*R* = 8.314 Pa m^3^/K/mol). *n*_t_^CO_2_^ is the known amount of CO_2_ present in the gas phase (RC + SC) at the beginning of the adsorption experiment (mol).

In this study, the adsorption experiments were conducted by increasing the CO_2_ pressure in stages, starting at 0.5 MPa until it reached to a maximum target pressure of 6.5 MPa pressure. CO_2_ desorption experiments were conducted using a pressure step-down procedure from the peak equilibrium pressure to the null pressure. Changes in gas phase pressure were recorded every 10 s to capture the rapid rate of adsorption–desorption at the beginning of the experiment and used to determine the adsorption capacity and kinetics. The experimental program is summarized in Table 2.

**Table 2:** Experimental Program of CO_2_ Adsorption and Desorption Tests Conducted in This Study^a^

	coal sample/location	9 ft AB		18 ft AB		BP	
pressure range	sample description	powder	intact	powder	intact	powder	intact
subcritical pressure (<6.1 MPa)	Adsorption test	√	√	√	√	√	√
	Desorption test	-	√	√	√	-	-
up to near-critical pressure (6.1–6.5 MPa)	Adsorption test	-	-	√	√	-	-
	Desorption test	-	-	√	√	-	-

a The tick mark represents the experiments conducted.

The van der Waals loop occurs near the critical pressure range (6.1 to 6.4 MPa at 298.15 K), in which liquid and vapor CO_2_ coexist and differ in molar volumes. The CO_2_ critical pressure value is 6.4 MPa at 298.15 K.^51^ Above this point, the coexistence of liquid and vapor is impossible. This is a key technical aspect that affects adsorption results as an overestimation or underestimation of CO_2_ excess adsorption calculations. To overcome this phenomenon, the following theoretical consideration was made in the current study to calculate the amount of CO_2_ adsorbed in the near critical region (6.1 MPa to 6.4 MPa at 298.15 K). When the vapor and liquid phase is in equilibrium, the chemical potential and Gibbs free energy of both phases are equal for pure fluids, and they are thermally and physically in equilibrium. The calculated molar volumes of liquid (*n*_L_) and vapor (*n*_v_) phases by PR-EoS at a given temperature and pressure were used to calculate the volume fraction (*v*^F^) of vapor phase CO_2_ following:^22,52^
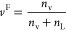
6where *n*_L_ and *n*_*v*_ are the molar volumes of liquid and vapor phases, respectively, and they are calculated using PR-EoS for a given pressure and temperature. The volume fraction ratio of vapor (*v*^F^) and liquid CO_2_ was used to calculate the total number of moles injected at near critical phase region (6.1 MPa to 6.4 MPa at 298.15 K).

## 3 Theory

### 3.1 Evaluation of CO_2_ Adsorption by Langmuir and BET Isotherm Models

The nonlinear form of the Langmuir isotherm model is expressed as^47,53−55^
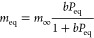
7where *P*_eq_ is equilibrium pressure (Pa), *m*_eq_ is the mass of CO_2_ adsorbed at given equilibrium pressure (g/kg), *m*_*∞*_ is limiting value of mass adsorbed (maximum adsorption capacity), also mass of a maximum monolayer adsorbate covering the surface of the sorbent (g/kg), and *b* is the Langmuir parameter, which is also reciprocal of half-loading pressure, (Pa^–1^). In this study, *m*_∞_ and *b* values were obtained from nonlinear regression analysis.

The thermodynamic parameters, e.g., energy of adsorption and Gibbs free energy were calculated from the Langmuir parameters (*m*_∞_ and *b*) as^56−58^
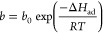
8
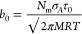
9where *b*_0_ is the exponential factor (Pa^–1^), Δ*H*_ad_ is energy of adsorption (J/mol), τ_0_ is vibration period related to the residence time of the adsorbed CO_2_ molecule (typically on the order of 10^–13^ s), *N*_m_ is the number of molecules adsorbed (related to m_∞_ and Avogadro’s number), σ_A_ is cross sectional area covered by one CO_2_ molecule (m^2^), *M* is molecular mass of CO_2_ (0.04401 kg/mol).

The Gibbs free energy (Δ*G*_ad_^0^, kJ/mol) can be calculated as^56^
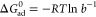
10The nonlinear form of the BET model is expressed as^59^
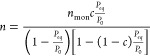
11where *n* is the amount of gas adsorbed (mol), *n*_mon_ is number of moles to cover monolayer adsorption (mol), *P*_eq_ is the equilibrium pressure (Pa), *P*_0_ is the saturation pressure (7.39 × 10^6^ Pa), *c* is dimensionless parameter related to energy of adsorption Δ*H*_ad_. The *c* and *n*_mon_ values were obtained from nonlinear regression analysis. The dimensionless parameter *c* is related to the adsorption energy and is defined as^59^
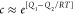
12where *Q*_1_ is the adsorption energy on a bare surface (monolayer adsorption) (J/mol), and *Q*_2_ is the energy of second and subsequent layers (J/mol).

The surface area of 1 mol of CO_2_ in the liquid state is calculated as^60,61^
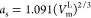
13
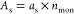
14where *a*_s_ is effective surface area covered by 1 mol of CO_2_ (m^2^/mol), and *V*_m_^L^ is liquid molar volume of CO_2_ (m^3^/mol). The number 1.091 is the packing factor of 12 neighboring molecules in a bulk liquid and six on a plane.^60^*A*_s_ is the specific surface area (m^2^/kg), and *n*_mon_ is number of moles required to complete the monolayer coverage per kg of coal sample, mol/kg.

### 3.2 Adsorption–Desorption Kinetics

The data obtained from the experimental program (Table 2) were fitted into pseudo-first-order (PSO) and pseudo-second-order rate equations.^62,63^
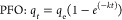
15
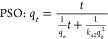
16where *q*_*t*_ represents the mass of CO_2_ adsorbed per unit mass of adsorbent at time *t* (g of CO_2_/kg of coal), *q*_e_ represents the mass CO_2_ adsorbed per unit mass of adsorbent at equilibrium (g of CO_2_/kg of coal), *k* is the first-order rate constant (h^–1^), and *k*_2_ is the second-order rate constants (kg g^–1^ h^–1^).

Desorption kinetics data were fitted into modified PFO and PSO equations. The equations are modified on the basis that the amount of CO_2_ adsorbed on coal is the rate determining factor.^36^
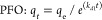
17
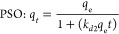
18where *k*_d1_ is the first-order rate constant for desorption, (h^–1^) and *k*_*d*2_ is the second-order rate constants for desorption (kg g^–1^ h^–1^).

To predict the influence of pore diffusion mechanism of coal-CO_2_ interaction, experimental data were also fitted into the Bangham model.^64−66^
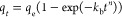
19where *k*_b_ (h^–1^) and *n* are constants of the model.

## 4 Results and Discussion

### 4.1 Adsorption Capacity of Intact and Powdered Coals at Subcritical and near Critical Conditions

**Figure 3:**
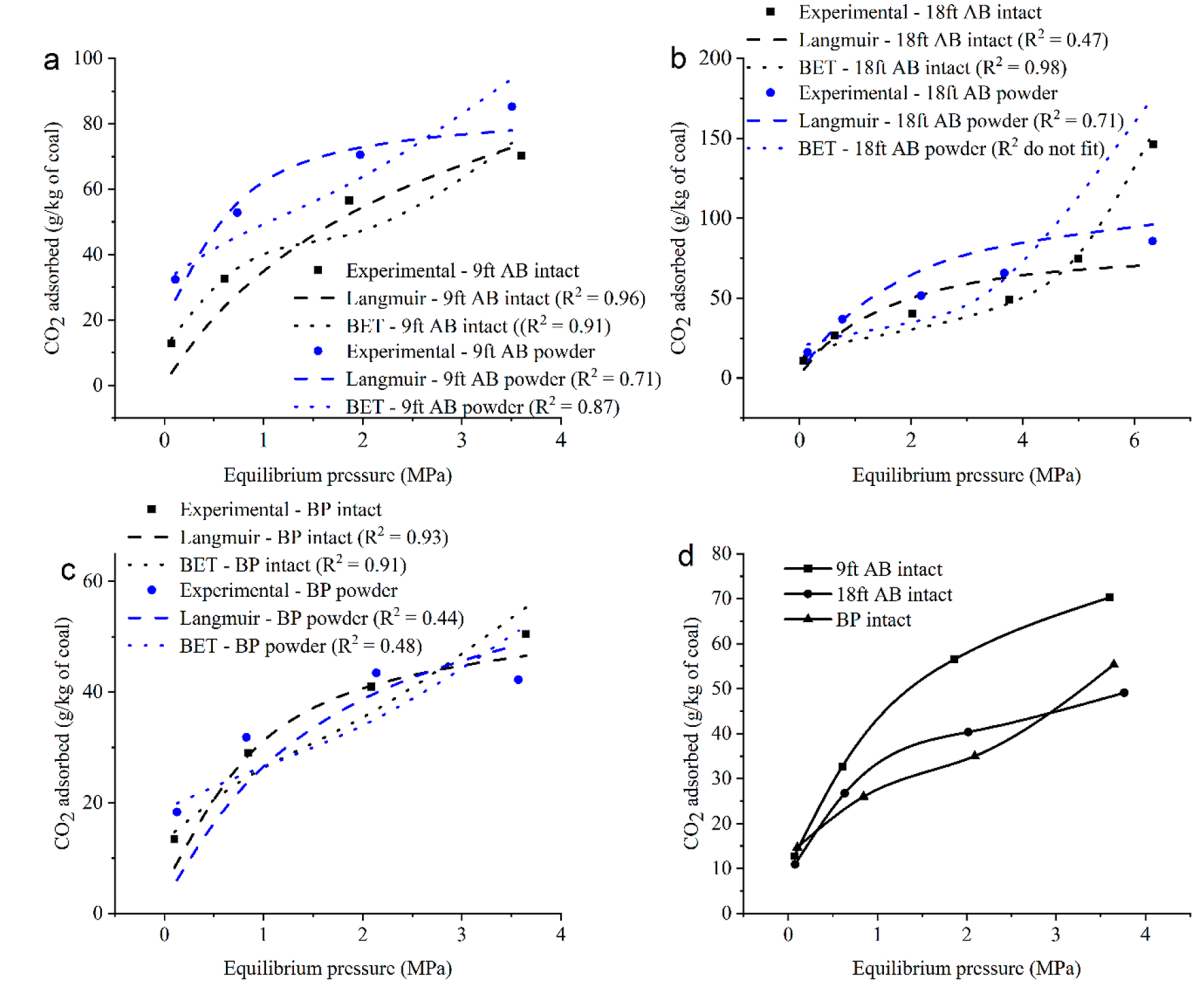
Experimental, Langmuir, and BET CO_2_ adsorption isotherms of (a) 9 ft Aberpergwm coal samples (powder and intact), (b) 18 ft Aberpergwm coal samples (powder and intact), (c) CO_2_ Big Pit coal (powder and intact), and (d) comparison of intact samples of 9 ft Aberpergwm, 18 ft Aberpergwm, and Big Pit coal.

Adsorption behavior of powder and intact samples of two different ranks are compared here. The adsorption isotherms of 9 ft AB, 18 ft AB, and BP coal samples are presented in Figure 3(a), (b), and (c), respectively for the maximum CO_2_ injection pressure up to 6.4 MPa, which is below the critical pressure of CO_2_ at 298.15 K. The powder samples of 9 and 18 ft AB anthracite coal exhibited greater CO_2_ adsorption than the intact samples (Figure 3a,b). In contrast, the intact bituminous sample of Big Pit coal exhibited similar adsorption behavior to that of the powder sample (Figure 3c). All the three intact coal samples showed similar adsorption isotherm pattern with 9 ft AB showing slightly higher adsorption capacity (Figure 3d).

The adsorption capacities of powder samples of the 9 and 18 ft AB coal were about 21% and 36% higher than that of intact samples, respectively. The results show that more adsorption sites are available in the powdered samples compared with the intact samples. The case is different when comparing powder and intact for bituminous BP samples. The CO_2_ adsorption isotherm pattern of the intact sample was similar to that of powdered samples for experiments conducted up to equilibrium pressures of 3.6 MPa (Figure 3c). Similar behavior for bituminous coal has been reported by Pone et al. (2009),^67^ where the adsorption capacity of powdered sample was 14% lower than the intact sample at applied pressure of 3.1 MPa. Zhao et al. (2014)^68^ reported that bituminous coal has channel-like and interconnected pores. Such pore structures are observed for both high- and low-volatile bituminous coals. Xu et al. (2015)^26^ and Tan et al. (2018)^27^ suggested that during the sample grinding process, powdered samples lost most of the channel-like fracture network and pore entrance. The porous matrix difference between the intact and powdered samples resulted in comparable adsorption capacities for the bituminous coal.

**Figure 4:**
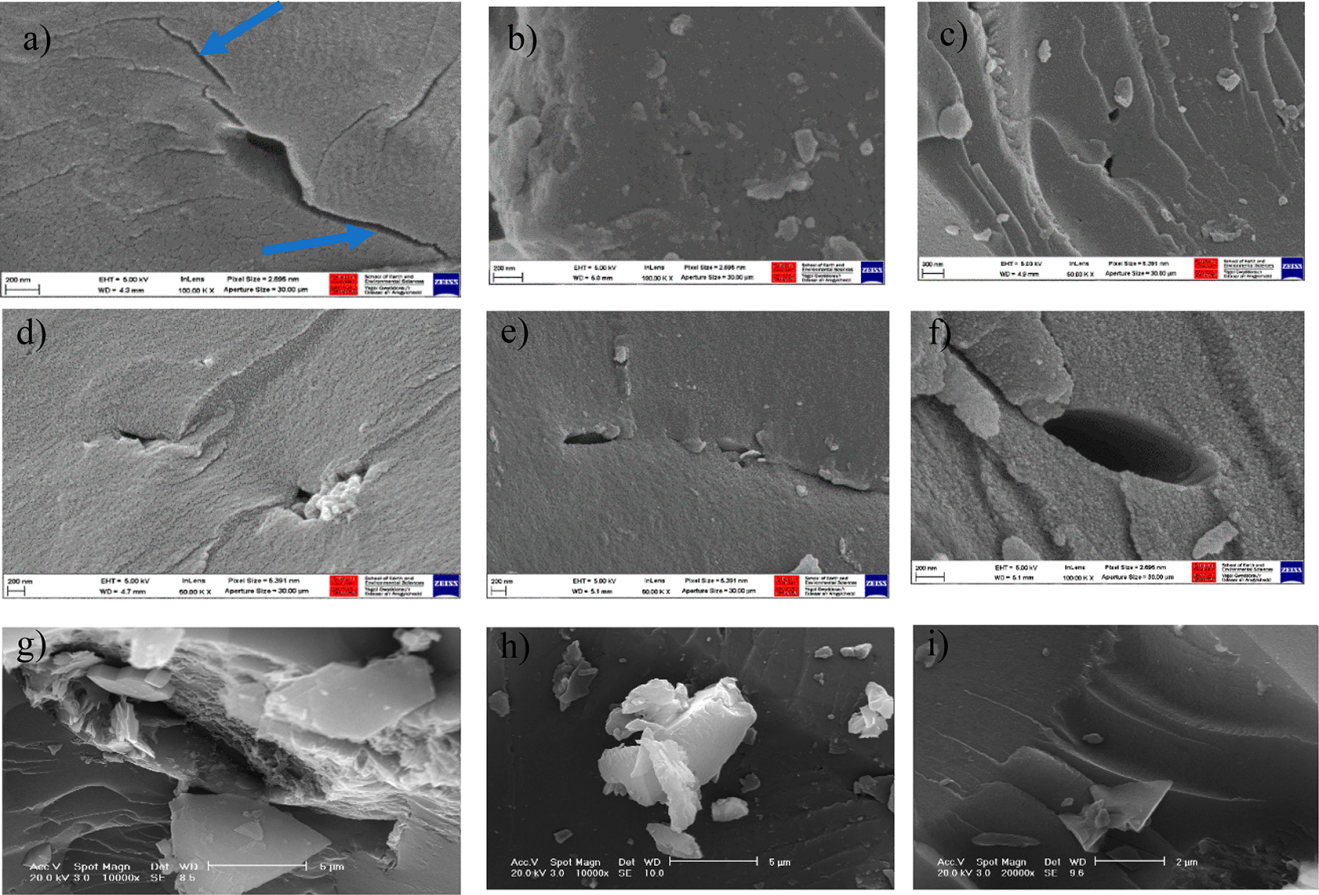
SEM images of a) BP bituminous intact coal, b, c) BP bituminous powder coal, d) 18 ft AB anthracite intact coal, e and f) 18 ft AB anthracite powder coal, and g) 9 ft AB anthracite intact coal, h) and i) 9 ft AB anthracite powder coal.

This analysis indicated that the sample physical nature has an impact on the CO_2_ adsorption on coal samples. To visualize the changes in sample structure, the SEM images of the powdered coal samples and intact samples were taken to identify the nano-/micropores. Figure 4 presents the SEM images for BP and 18 ft AB intact and powdered samples. The nanosized channel like pore entrance/fractures are clearly visible in the intact specimens of the bituminous sample (Figure 4a). However, the channel like pore openings have not been identified in the powdered bituminous BP sample indicating the pulverization of the sample destroys them (Figure 4b,c). The features of intact bituminous sample have not been identified in anthracite intact samples (Figure 4d). The anthracite 18 ft AB powdered samples showed the exposed nanosized pore entrances (Figure 4e,f) which were observed in intact samples as well. Similarly, no discernible differences were found between anthracite 9 ft AB intact and powdered samples (Figure 4g–i). These results are consistent with the adsorption isotherm pattern observed for the intact and powdered samples of the bituminous and anthracite coal samples. As discussed elsewhere, the high-density CO_2_ adsorption in channel-like pores of the intact bituminous coal reflected the adsorption capacity (Figure 3).

The results from the adsorption tests and SEM images clearly showed that the powdered and intact samples of coals considered in this investigation exhibited different adsorption capacities. Therefore, testing intact samples is more appropriate for determining the adsorption capacity since it reflects the fabric and structure of in situ coal seam.

The injection pressure is considered as one of the key parameters that affects the adsorption capacity of coals. For pressures lower than 6.1 MPa, only the gas phase would exist in the system. At higher pressures, that is, near the critical region (6.1 to 6.4 MPa), the coexistence of liquid and vapor phases of CO_2_ is evident. The fraction of liquid and gas CO_2_ coexisted during the injection were calculated as described in section 2.3.

The adsorption isotherms of 18 ft AB powder and intact coal for an injection pressure range of 0.5 to 6.4 MPa are presented in Figure 3b. It can be seen from Figure 3b that the 18 ft AB intact specimen showed maximum capacity of 3.328 mol of CO_2_/kg of coal (146 g/kg), and the powdered specimen exhibited 1.843 mol of CO_2_/kg of coal (52.12 g/kg). It is noted that, at lower pressures (<6.1 MPa), the powdered sample showed a higher adsorption capacity than the intact sample. However, at a near critical injection pressure range (6.1 to 6.4 MPa), the intact sample showed a higher adsorption capacity than the powder. This is because the condensation and high-density CO_2_ adsorption occur in the nanopores and microfractures of intact samples due to the gas cavitation or condensation.

### 4.2 Isotherm Modeling Results

#### 4.2.1 Evaluation of CO_2_ Adsorption on Coal Using the Langmuir Model

To validate the model, the CO_2_ adsorption equilibrium data from the experiments were fitted with the mathematical expression of the Langmuir model using nonlinear regression analysis (eq 7) and are shown in Figure 3a–c and Table 3. Figure 3a–c shows the plots comparing the experimental data of intact and powdered samples of 9 ft AB, 18 ft AB, and BP against results obtained from the Langmuir model. Table 3 summarizes the Langmuir parameters.

**Table 3:** Langmuir Parameters, *b* - Half-Loading Pressure, and *m*_∞_ - Maximum Adsorption Capacity, Obtained from Plots (Figure 3)

sample description	half-loading parameter *b*, Pa^**–**1^	maximum adsorption capacity, *m*_**∞**_, g of CO_2_/kg of coal	*R*^2^ and (standard error of estimate)
AB 18 ft intact	8.50 × 10^–7^	84.00	0.47 (32)
AB 18 ft powder	6.61 × 10^–7^	119.00	0.71 (13)
AB 9 ft intact	1.56 × 10^–6^	80.54	0.96 (4.5)
AB 9 ft powder	2.49 × 10^–6^	91.77	0.71 (10.6)
Big Pit intact	1.80 × 10^–6^	53.66	0.93 (4.1)
Big Pit powder	4.29 × 10^–6^	45.57	0.44 (7.5)

At lower and intermediate pressures (<6.1 MPa), there was good agreement between experimental and model results (Figure 3a,c). The experimental results deviated from the model to show the multilayer build-up at high pressures (from 6.1 to 6.4 MPa). This was evident in the intact and powdered samples of 18 ft AB (Figure 3b). These findings support the expected theory of high-density CO_2_ adsorption in microporous structure of the intact samples at elevated pressures.

It was explained in the previous section why the bituminous intact coal sample had a similar adsorption capacity as the powdered sample of the same coal, which was reflected in the calculated maximum capacity. The predicted Langmuir maximum adsorption capacity for the BP intact (bituminous) was 53.66 g of CO_2_/kg of coal, while the powdered BP sample was 45.57 g of CO_2_/kg of coal. The inverse of half-loading pressure of all the samples (Langmuir parameter *b*, Pa^–1^), the predicted pressure at which half of the maximum adsorption capacity can be achieved, ranged from 10^–6^ to 10^–7^ Pa^–1^ (Table 5).

Half-loading pressure is an important economic parameter in coal seam CO_2_ storage.^69^ Conducting an isobaric adsorption experiment at the half-loading pressure value predicted by the Langmuir model can yield half of the maximum adsorption capacity of the specific coal sample. Experiments at 1.59 MPa (half-loading pressure; reciprocal of the *b*-value for 18 ft AB intact) can, for example, achieve a loading of half the 86.97 g CO_2_/kg of coal (Figure 3b; Table 3). Similarly, at 0.40 MPa (inverse of the *b* value for 9 ft AB intact; Figure 3a; Table 3), half of 80.54 g of CO_2_/kg of coal can be loaded.

The observations discussed above imply that the monolayer was covered at pressures less than 1 MPa, and the isotherm pattern observed at intermediate pressures showed the type II isotherm slope^70^ rather than the plateau typically observed in Langmuir type adsorption, representing the multilayer build-up that occurs at coal surfaces.^60^ The pattern was visible in the intact coal samples because capillary condensation occurs in the pores of the intact samples, and the plots in Figure 3a–c showed a deviating uptrend from the Langmuir model.

The adsorption energy (Δ*H*_ad_) was calculated using the Langmuir parameters obtained from the isotherm model fittings (maximum Langmuir adsorption capacity (*m*_∞_) and the constant *b*). The energy of adsorption values was between −15 kJ/mol and −22 kJ/mol (Table 4), which are attributed to the physical adsorption (enthalpy change is in the range of −20–40 kJ/mol^71^).

**Table 4:** Estimated Values of Energy of Adsorption (Based on Langmuir Parameters and the Kinetic Theory of Gases

sample description	Δ*H*_ad_, kJ/mol	Δ*G*_ad_^0^, kJ/mol
18 ft intact	–16.38	–34.65
18 ft powder	–14.89	–35.27
AB 9 ft intact	–17.99	–33.14
AB 9 ft powder	–18.68	–31.98
Big Pit intact	–19.36	–32.8
Big Pit powder	–21.91	–30.64

The Gibbs free energy of CO_2_ adsorption on coal was calculated and is shown in Table 4. The adsorption energy/Gibbs free energy of adsorption are molar quantities that increase with the number of moles adsorbed. The findings were directly compared with previously reported findings on an 86% carbon content coal, where the enthalpy of adsorption ranged from 25.3 to 27.3 kJ/mol,^72^ which is comparable with the current study’s estimated values. The adsorption energy calculated in this work is similar to the numerical values of the heat of condensation of CO_2_ (15.8 kJ/mol^73^), indicating that liquid-like adsorption theories such as BET should be used to explain CO_2_ adsorption on coal.

#### 4.2.2 Evaluation of CO_2_ Adsorption on Coal Using Brunauer–Emmet–Teller (BET) Model

Figure 3a–c shows plots comparing experimental data from intact and powdered samples of 9 ft AB, 18 ft AB, and BP to results from the BET model. The BET model fitted the experimental data well (Figure 3a–c), indicating multilayer CO_2_ adsorption on coal surfaces. Table 5 provides a summary of the BET parameters of the coal samples.

**Table 5:** Brunauer–Emmet–Teller (BET) Parameters of CO_2_ Adsorption on Coal Samples

sample description	BET dimensionless parameter, *c*	adsorbed amount at monolayer coverage, *N*_m_, g of CO_2_/kg of coal	*R*^2^ and (standard error of estimate)	specific surface area, m^2^/kg	*Q*_1_ – *Q*_2_, (kJ/mol)^a^
18 ft AB intact	150	21.96	0.98 (6.6)	78408	12.42
18 ft AB powder	222	25.17	–0.2 (42)	89869	13.39
9 ft AB intact	52.95	32.79	0.91(6.5)	121397	9.839
9 ft AB powder	209	42.87	0.87 (7.1)	153067	13.24
Big pit intact	93.5	24.16	0.91 (4.1)	86263	11.25
Big pit powder	296.45	22.80	0.48 (7.2)	81407	14.11

a *Q*_1_ – *Q*_2_ is the difference between the energy of adsorption of the first layer and the subsequent liquid layers.

The monolayer coverage, *N*_m_, values show that the inflection point occurred well below 1.0 MPa (Figure 3a–c; Table 5). This could be the reasons that the intermediate pressure experiments fit the BET model well where the liquid like adsorption occurs above the inflection point.

*Q*_1_ kJ/mol (Table 5) is related to the energy of first layer adsorption (Δ*H*_ad_). The magnitude of *Q*_1_ – *Q*_2_ is related to the heat of condensation or liquefaction of CO_2_. The energy of adsorption values observed in the current study was comparable with the heat of condensation of CO_2_ (approximately −16.7 kJ/mol^74^). This analysis indicates that CO_2_ pore condensation occurs above the inflection point (>1 MPa; Figure 3a–c).

Table 5 summarizes the calculated available specific surface area on coal for the adsorption of gas molecules. The intact sample of bituminous BP coal (86263 m^2^/kg) showed a higher surface area than the intact samples of anthracite 18 ft AB (78408 m^2^/kg) and lower than the 9 ft AB (121397 m^2^/kg) samples. The powdered samples of 9 ft AB had a higher specific surface area than the powdered samples of 18 ft AB and BP samples. The intact sample of BP (86263 m^2^/kg) exhibited a similar specific microporous surface as the powdered samples of BP (81407 m^2^/kg). This is effect of the bituminous coal structure (microporous porous network volume) on the adsorption of CO_2_ in intact bituminous sample which resulted in a higher adsorption capacity. The obtained specific surface areas in the current work (78408 m^2^/kg to 153067 m^2^/kg) are comparable with the specific surface area available for CO_2_ obtained by Zhao et al. (2016)^75^ (77400 m^2^/kg to 198400 m^2^/kg).

#### 4.2.3 Comparison of Langmuir and Brunauer– Emmet–Teller (BET) Models

To evaluate the adsorption of CO_2_ by the coal samples, the Langmuir and BET models were compared based on the experimental data fitting effect. Nonlinear regression analysis has been used to fit the theoretical models and experimental results. The optimal model is shown by the combination of the correlation coefficient (*R*^2^) and standard error of estimation (SEOE) (Tables 4 and 5). The BET model generally demonstrated good agreement with the experimental results for the intact samples. The experimental findings of powdered 18 ft AB and 9 ft AB anthracite samples fit the Langmuir model quite well. Both the BET and Langmuir models fit well with the experimental data of powder and intact samples of BP bituminous coal.

### 4.3 Kinetics of CO_2_ Adsorption on Different Condition and Rank of Coal

**Figure 5:**
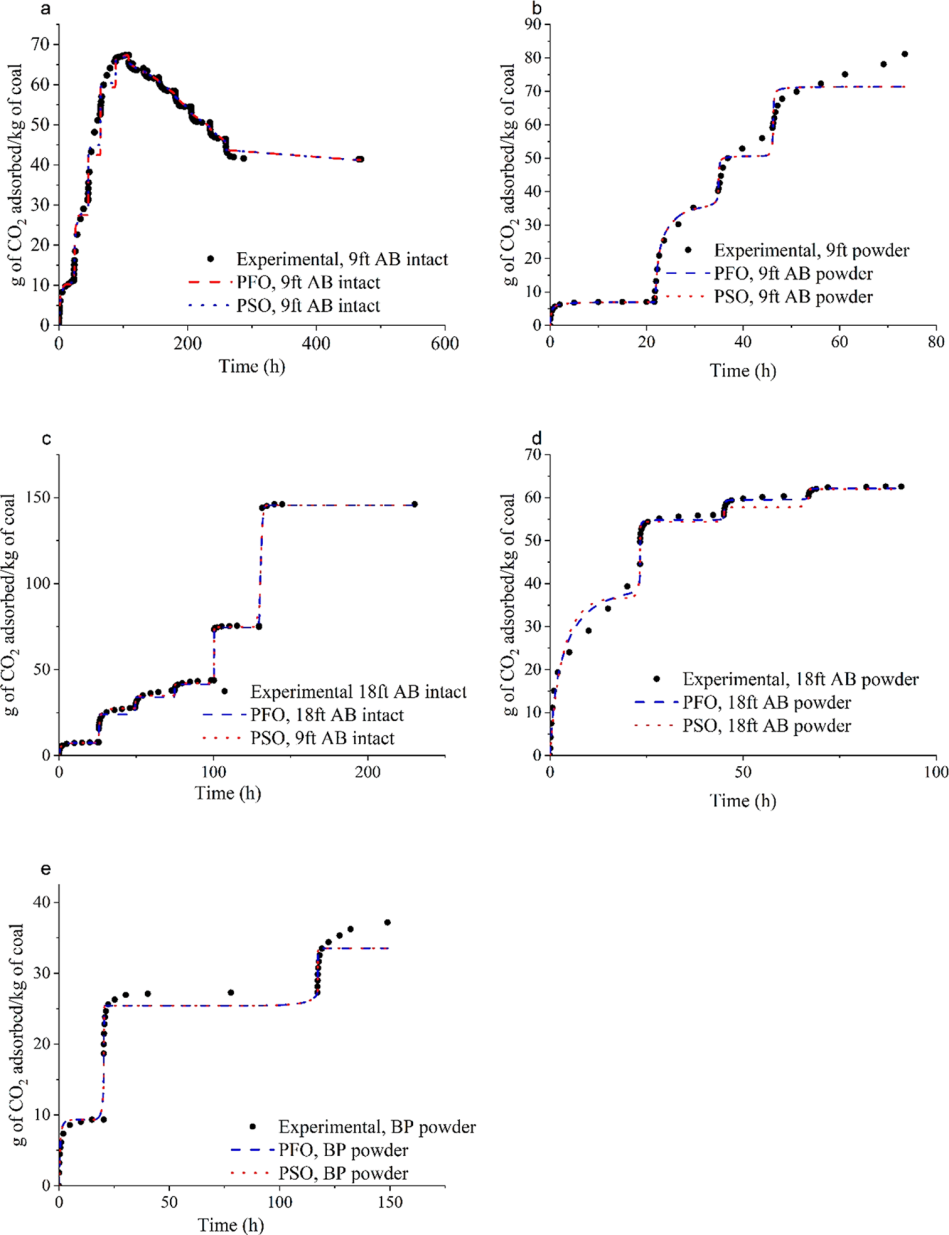
Experimental data fitted against the PFO and PSO model; (a) 9 ft AB intact, (b) 9 ft AB powder, (c) 18 ft AB intact, (d) 18 ft AB powder, and (e) Big Pit (BP) powder.

Pseudo-first order (PFO) and pseudo-second order (PSO) kinetic models were used to fit the experimental data (that is, the amount adsorbed CO_2_ versus time). The adsorbed CO_2_ predicted by the PFO and PSO models at time *t* (*q*_*t*_, g adsorbed CO_2_/kg coal) are plotted against the experimental values in Figure 5. Figure 5, panels a–e shows the results obtained for samples of 9 ft AB intact, 9 ft AB powder, 18 ft AB intact, 18 ft AB powder, and Big Pit powder, respectively. Tables 6, 7, and 8 show the rate constants (*K*_ad1_, *K*_ad2_ for adsorption, and *K*_de1_ for desorption), equilibrium concentration (*q*_e_), standard error of estimate and *R*^2^ values for the model fit for the 9 ft AB intact, 9 ft AB powder, 18 ft AB dry intact, 18 ft AB powder, and Big Pit powder, respectively.

**Table 6:** PSO and PFO Model Parameters Obtained for 9 ft AB Samples

		pseudo-first-order kinetics parameters			pseudo-second-order kinetics parameters		
	injection - equilibrium pressure (MPa)	equilibrium concentration (*q*_*e*_) g of CO_2_/kg of coal	rate constant, *k*_a1_ (h^–1^)	*R*^2^ and standard error of estimate	equilibrium concentration (q_e)_ g of CO_2_/kg of coal	rate constant *k*_a*2*_ (kg g^–1^ h^–1^)	*R*^2^ and (standard error of estimate)
Powder	Adsorption						
	0.75–0.11	6.72	2.87	0.92 (0.49)	6.96	0.7	0.99 (0.19)
	1.51–0.73	34.26	1.07	0.85 (3.89)	37.99	0.04	0.99 (2.85)
	3–1.97	50.98	9.24	0.35 (4.65)	50.74	1.27	0.99 (4.67)
	4.51–3.5	69.71	37.13	0.2 (6)	71.42	0.92	0.42 (5)
Intact	Adsorption						
	0.56–0.07	10.46	0.52	0.96 (0.84)	11.52	0.058	0.99 (0.48)
	1.54–0.6	26.63	1.54	0.50 (5.12)	27.96	0.09	0.53 (5.29)
	3.03–1.86	44.99	4.86	0.25 (7.24)	47.94	0.14	0.7 (4.56)
	4.51–3.6	58.71	953	0.01 (4.80)	60.41	1.54	0.45 (3.80)
	Desorption		*k*_d1_ (h^–1^)				
	3.06–3.23	66.81	102	0.17 (0.24)	66.88	30.17	0.35 (0.26)
	1.87–2.38	65.43	0.002	0.51 (0.74)	65.44	2.79 × 10^–5^	0.51 (0.74)
	1.53–1.89	66.39	0.002	0.66 (0.52)	63.4	2.54 × 10^–5^	0.66 (0.52)
	1–1.4	60.89	0.002	0.76 (0.62)	60.9	3.63 × 10^–5^	0.76 (0.61)
	0.6–0.99	57.06	0.0025	0.65 (0.86)	57.07	4.67 × 10^–5^	0.66 (0.87)
	0.31–0.66	52.84	0.002	0.51 (0.99)	52.86	4.33 × 10^–5^	0.52 (0.98)
	0.1–0.42	48.96	0.003	0.61 (0.84)	48.98	5.76 × 10^–5^	0.62 (0.91)
	0–0.22	43.73	0.0002	0.33 (1.4)	48.98	6.85 × 10^–5^	0.33 (1.4)

**Table 7:** PSO and PFO Model Parameters Obtained for 18 ft AB

		pseudo-first-order kinetics parameters			pseudo-second-order kinetics parameters		
	injection - equilibrium pressure (MPa)	*q*_e_, equilibrium concentration, g of CO_2_/kg of coal	rate constant, *k*_a1_ (h^–1^)	*R*^2^ and standard error of estimate	*q*_e_, equilibrium concentration, g of CO_2_/kg of coal	rate constant *k*_a2_, (kg g^**-**1^ h^–1^)	*R*^2^ and (standard error of estimate)
Intact	0.58–0.08	7.25	1.21	0.95 (0.5)	7.75	0.23	0.99 (0.23)
	1.53–0.63	23.97	17.64	0.49 (3.06)	27.73	0.19	0.99 (1.26)
	3.04–2.02	33.37	31.57	0.25 (2.82)	37.83	1.68	0.60 (2.26)
	4.59–3.76	41.36	410.9	0.02 (1.7)	42.04	4.14	0.55 (1.24)
	5.52–4.99	74.47	346.91	0.92 (0.77)	74.82	9.5	0.99 (0.52)
	6.35–6.33	145.54	346	0.99 (0.81)	145.54	346	0.99 (0.81)
Powder	0.68–0.15	36.68	0.35	0.97 (4.18)	42.08	0.01	0.95 (3.08)
	1.65–0.77	54.39	52.93	0.99 (1.33)	54.85	3.48	0.91 (0.95)
	3.22–2.18	57.75	78.8	0.99 (1.59)	59.54	5.45	0.99 (0.61)
	4.5–3.67	61.96	74.77	0.99 (0.35)	62.14	9.92	0.99 (0.49)

**Table 8:** PSO and PFO Model Parameters Obtained for Big Pit Coal

	pseudo-first-order kinetics parameters			pseudo-second-order kinetics parameters		
injection - equilibrium pressure (MPa)	*q*_*e*_, equilibrium concentration, g of CO_2_/kg of coal	rate constant, *k*_a1_, h^–1^	*R*^2^ and standard error of estimate	*q*_e_, equilibrium concentration, g of CO_2_/kg of coal	rate constant *k*_a2_, kg g^–1^ h^–1^	*R*^2^ and (standard error of estimate)
0.53–0.13	9.33	1.70	0.99 (0.88)	9.25	0.28	0.99 (0.39)
1.54–0.82	25.4	28.75	0.46 (1.73)	26.11	1.84	0.99 (1.13)
3.12–2.13	33.52	39.1	0.45 (2.12)	37.18	40.99	0.98 (4.5)

Overall, the plots and the combination of standard error of estimate and correlation coefficient (*R*^2^) indicate that the adsorption kinetics data agree well with the PSO model than the PFO model. The PSO model implies that surface interaction and bulk pore diffusion dominate CO_2_ adsorption on coal.^76,77^ The pressure dependence of *K*_ad2_ demonstrated that the pore diffusion being the rate-determining step in the beginning and surface interaction being the slowest rate-determining step at higher pressures (*K*_ad1_, *K*_ad2_ values in Tables 6–8). There are only a few other studies to compare the kinetic parameters with. Gabruś et al. (2021)^78^ published an experimental study on CO_2_ adsorption on bituminous coal in which the results have been fitted into PFO and PSO models. The *K*_ad1_ and *K*_ad2_ values obtained at 298.15 K and 2 MPa equilibrium pressure were much higher than the values obtained in the current study. *K*_ad1_ was in the range of 1613 × 10^3^ to 1011 × 10^3^ h^–1^, and *K*_ad2_ was in the range of 5752 × 10^3^ h^–1^ to 11851 × 10^3^ h^–1^. However, these experiments were conducted for less than 24 h equilibrium time to reach the maximum pressure range of 2 MPa whereas the current study allowed the equilibrium to occur for each pressure steps (0.5 to 6.4 MPa).

Pore diffusion was more pronounced during the desorption kinetics, which fits both the PFO and PSO models well. The rate-limiting step in the desorption process was the slow release of CO_2_ molecules trapped in the pores (*K*_de1_ values; Table 6). It can be seen from the results presented in Figure 5a that the adsorption–desorption kinetics plots of 9 ft AB intact, the model plot fitted very well with PFO model implying that the rate of desorption depends on the CO_2_ trapped in the pores.

**Figure 6:**
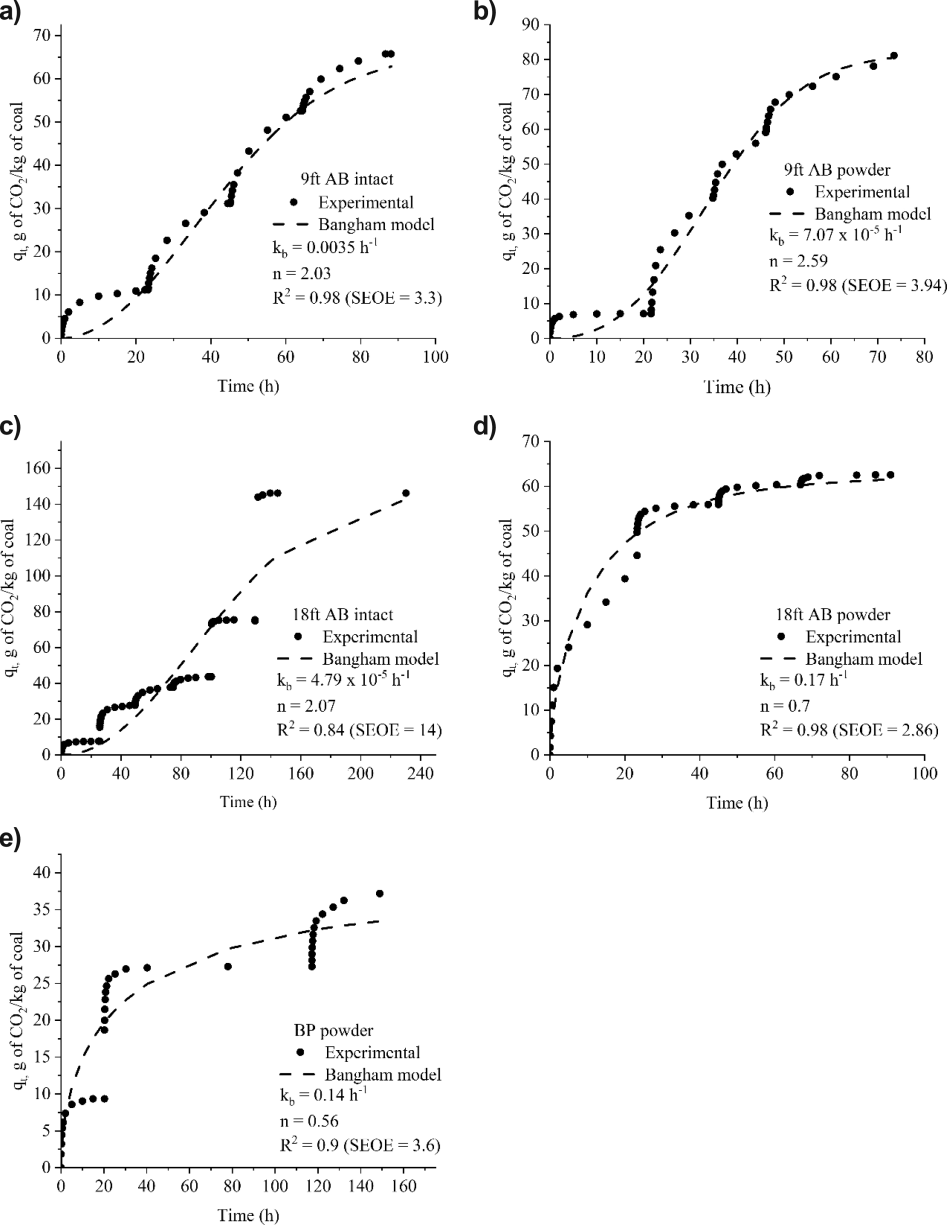
Bangham kinetic pore diffusion model fitting for (a) 9 ft AB intact (pressure range for up to 6.3 MPa), (b) 9 ft AB powder (pressure range for up to 6.3 MPa), (c) 18 ft AB intact (pressure range for up to 6.3 MPa), (d) 18 ft AB powder (pressure range for up to 6.3 MPa), and (e) BP powder (pressure range for up to 6.3 MPa).

In order to explore whether the CO_2_ adsorption is influenced by the mass transport phenomena of pore diffusion, the adsorption experimental data of 9 ft AB the Bangham model were used (eq 19).^37,65^ The Bangham model assumes that pore diffusion influences the kinetics of the adsorption process. Equation 19 presents the nonlinear form of the model. The correlation coefficient (*R*^2^) from the best-fit indicates the pore diffusion and pressure dependency of the constants *k*_b_ (h^–1^), and *n* indicates the rate-determining step at the corresponding pressure range. The kinetic data acquired for the intact and powdered samples of 9 ft AB 18 ft AB, and powdered samples of BP were fitted with the model. Figure 6, panels a–e presents results for 9 ft AB intact, 9 ft AB powder, 18 ft AB intact, 18 ft AB powder, and Big Pit powder, respectively.

The higher correlation coefficient (*R*^2^) in Figure 6a–e show that the pore diffusion is one of the rate determining steps. The correlation coefficient (*R*^2^) value obtained for high pressure experiments up to 6.3 MPa for 18 ft AB coal was 0.84 (Figure 6c), which was less than the values obtained for lower pressure experiments for 18 ft AB powder (*R*^2^ = 0.98; Figure 6d), 9 ft AB intact (*R*^2^ = 0.98; Figure 6a), 9 ft AB powder (*R*^2^ = 0.98; Figure 6b), and Big Pit powder (*R*^2^ = 0.9; Figure 6e). These findings indicate that at lower pressures, bulk pore diffusion is the primary rate-determining step, while at higher pressures, surface interaction takes over, which is the slowest step.^79^ Overall, the experimental data obtained from the current study fitted very well with the PSO kinetic model and Bangham pore diffusion model indicating that surface interaction and pore diffusion/condensation are the rate-determining steps in the CO_2_ adsorption process on coal. The PSO model fit the data better than the Bangham model (based on *R*^2^ and SEOE). However, the PSO model required separate segment fitting of each experimental pressure step up stage, whereas the Bangham model can use the entire data set without fitting each pressure step up stage separately.

### 4.4 Adsorption–Desorption Hysteresis

**Figure 7:**
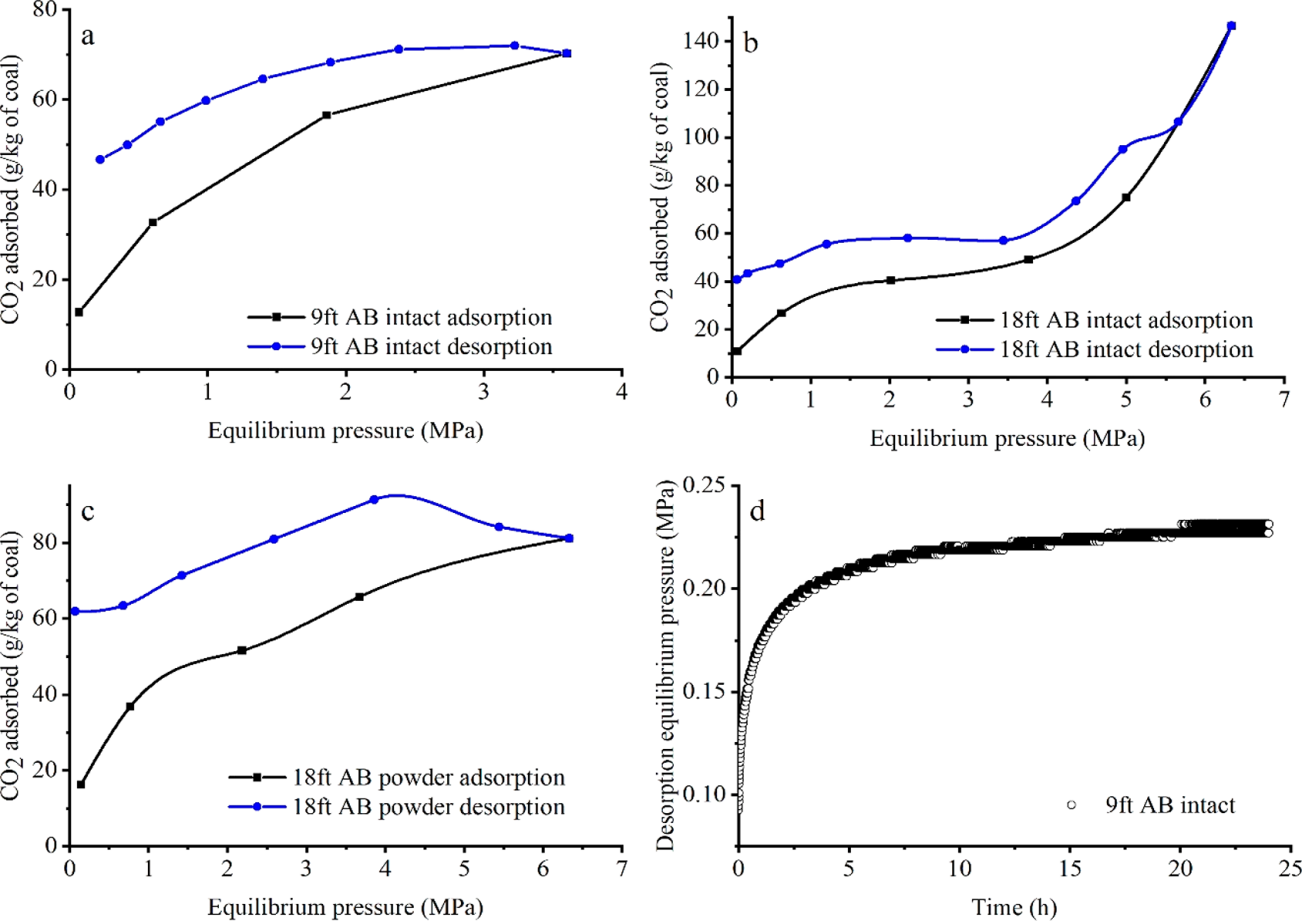
CO_2_ adsorption and desorption behavior of (a) intact 9 ft Aberpergwm coal, (b) intact 18 ft Aberpergwm coal, (c) powdered 18 ft Aberpergwm, (d) pressure versus time curves observed for intact 9 ft AB sample after evacuating the sample to 0 MPa at 298.15 K.

The adsorption and desorption isotherms of intact 9 ft AB, intact 18 ft AB, and powdered 18 ft AB coal samples are presented in Figure 7a,b,d. The positive deviation in the hysteresis indicates that some amount of CO_2_ still adsorbed in the porous structure of the coal. This pattern also depicts the Type II and H3 adsorption–desorption pattern described by IUPAC classification for the pore diffusion/condensation dominated adsorption process.^70,80^

Adsorption hysteresis patterns differ between powder and intact samples of 18 ft AB for experiments up to 6.4 MPa, owing to high-density adsorbed phase formation in the intact coal fracture system and micropores. Comparatively the lower adsorption capacity observed with powdered 18 ft AB shows the effect of the physical nature of the intact sample and the effect of near critical pressure range (6.1 to 6.4 MPa; liquid and gas coexistence) CO_2_ adsorption at 298.15 K (Figure 7b,c).

The adsorption–desorption hysteresis patterns were found to be consistent with previous work.^69,81−85^ The current study experiments and findings reveal that the physical nature of coal and the thermodynamic nature of CO_2_ provide a pathway to a deep pore matrix in which CO_2_ molecules are trapped. Because CO_2_ can enter through supermicropores (<2 nm), the ink bottle effect was observed in the hysteresis pattern of both intact and powdered samples.^29^ As a result, the slow release of CO_2_ trapped in the pores is observed as a positive deviation in the adsorption–desorption hysteresis pattern.

The amount of CO_2_ that remained in the coal after the desorption experiments demonstrated the coal seams’ ability to trap CO_2_. The residual values for intact 9 ft AB, intact 18 ft AB, and powder 18 ft AB coal samples were 46.21 g/kg (1.05 mol/kg), 39.61 g/kg (0.9 mol/kg), and 51.93 g/kg (1.18 mol/kg), respectively. The higher residual amount of CO_2_ adsorbed for the powdered sample for the 18 ft AB was attributed to the adsorption of CO_2_ in the exposed (when the samples are powdered) super micropores (<2 nm) and evidence of ink bottle effect.^83^Figure 7d shows pressure versus time curves observed for intact 9 ft AB sample after evacuating the whole adsorption system to create null equilibrium pressure (0 MPa) at 298.15 K indicating the CO_2_ entrapment in the pores.

The reversibility of CO_2_ and the adsorption desorption hysteresis pattern are influenced by the sample type (intact and powdered) and coal rank. The hysteresis index (HI) values were calculated for 9 ft AB and 18 ft AB coal samples to evaluate the adsorption–desorption as described in Wang et al. (2014)^86^ and Wang et al. (2016).^87^ The adsorption desorption is completely reversible when HI = 0 and irreversible when HI = 1. Comparing the anthracite 18 ft AB intact and powder, the powder sample showed higher HI values (0.92) than that of intact samples (0.16). However, the low carbon content intact anthracitic sample (9 ft AB) had HI = 0.70. The carbon content is related to the coal rank and pore structure reflected on the desorption pattern. The higher HI (0.92) obtained for the higher rank powdered anthracitic 18 ft AB showed that the nanopores are exposed to CO_2_ to enter when powdered indicating the sample physical type plays critical role in the desorption patten.

## 5 Conclusions

The adsorption–desorption isotherm patterns and kinetics of powdered and intact specimens of anthracite (9 ft AB and 18 ft AB) and bituminous (BP) coals obtained at the subcritical pressure range (up to 6.4 MPa) of CO_2_ at temperature of 298.15 K using the manometric/volumetric adsorption measurement method were presented in this study.

- The CO_2_ adsorption capacity of powdered samples of anthracite samples showed a higher adsorption capacity than that of intact samples due to the increased surface area which exposes the polarizing sites of anthracite coal and the nanopores.
- The intact bituminous (BP) coal showed a similar adsorption capacity as the powdered samples. Pulverizing the sample destroys the microfracture channel-like network that is specific to bituminous coals, and the results observed in this study demonstrated the effect of different porous networks of anthracite and bituminous coal samples on the CO_2_ adsorption capacity.
- The experiments conducted at the near critical region (up to 6.4 MPa) showed that the intact samples exhibited a type II adsorption isotherm pattern with an upward trend, and the powdered sample showed a monolayer type plateau indicating the sample’s physical nature had an impact on the type of the isotherm that occurs.
- Overall, the fitting of the adsorption experimental data and the theoretical model in this paper show that the BET model fits better than the Langmuir model.
- Within the coal rank, the reversibility of the CO_2_ and hysteresis pattern were affected by the sample type (intact and powder). The powdered samples of anthracitic sample showed a higher degree CO_2_ irreversibility than the intact sample indicating the CO_2_ entrapment in the exposed nanopores. The analysis indicated that the experiments with large undisturbed samples are needed to test the adsorption capacity of specific coal type. The experimental results were fitted to PFO, PSO, and Bangham pore diffusion kinetic models, which showed that surface interaction and pore diffusion mechanisms are the rate-determining mechanisms of CO_2_-coal adsorption processes.

Overall, the paper explored the effect of physical nature and subcritical pressure adsorption of CO_2_ at 298.15 K in the context of CO_2_ sequestration in shallow level coal seams.
